# An Optimalization Study on the Surface Texture and Machining Parameters of 60CrMoV18-5 Steel by EDM †

**DOI:** 10.3390/ma15103559

**Published:** 2022-05-16

**Authors:** Panagiotis Karmiris-Obratański, Emmanouil L. Papazoglou, Beata Leszczyńska-Madej, Nikolaos E. Karkalos, Angelos P. Markopoulos

**Affiliations:** 1 Department of Manufacturing Systems, Faculty of Mechanical Engineering and Robotics, AGH University of Science and Technology, 30-059 Cracow, Poland; 2 Laboratory of Manufacturing Technology, School of Mechanical Engineering, National Technical University of Athens, 15780 Athens, Greece; mlpapazoglou@mail.ntua.gr (E.L.P.); nkark@mail.ntua.gr (N.E.K.); amark@mail.ntua.gr (A.P.M.); 3 Department of Materials Science and Non-Ferrous Metals Engineering, Faculty of Non-Ferrous Metals, AGH University of Science and Technology, 30-059 Cracow, Poland; bleszcz@agh.edu.pl

**Keywords:** EDM, CALMAX, Taguchi DOE, ANOVA, grey relational analysis, white layer formation

## Abstract

As a non-conventional machining technology, EDM is used extensively in modern industry, particularly in machining difficult-to-cut materials. CALMAX is a chromium-molybdenum-vanadium tool steel with exceptional toughness, ductility, and wear resistance that has a wide range of applications. Despite the fact that EDM is routinely used in CALMAX machining, the related published research is brief and limited. The current research gives a complete experimental study of CALMAX machining using EDM. A Taguchi Design of Experiment (DOE) was used, using pulse-on current, pulse-on time, and open-circuit voltage as control parameters. Material Removal Rate (MRR), Tool Material Removal Rate (TMRR), and Tool Wear Ratio (TWR) were used to evaluate machining performance, while Ra and Rz were used to estimate Surface Quality (SQ). The produced White Layer (WL) parameters were determined using optical and SEM microscopy, as well as EDX measurements and micro-hardness studies. Finally, for each of the aforementioned indexes, Analysis of Variance (ANOVA) was performed, and multi-objective optimization was based on Grey Relational Analysis (GRA). The results show that higher open-circuit voltage produces lower WL thickness, although by increasing the pulse-on time, the TWR is increased. The average hardness of the WL is increased about 400% compared to the micro-hardness of the bulk material.

## 1. Introduction

Electrical discharge machining is a non-conventional machining process suited for processing electrically conductive materials, regardless of their hardness, strength, or other mechanical properties. By employing EDM, complex shapes and geometries with a high level of dimensional accuracy and surface quality can be manufactured [1]. Conceptually, in EDM, the material removal is resulted due to repetitive, precisely controlled sparks occurring between the working electrode and the workpiece in the presence of a dielectric fluid. During the spark, a tiny topical plasma channel is formed, with temperatures reaching up to 12,000 °C, resulting in the melting of the material and/or ablation. The total material removal accumulates thousands or even millions of successive sparks [2]. The inherent advantages of EDM render it a feasible process, widely utilized in a modern industrial environment, namely, in automotive and aerospace industry, for tool and molds manufacturing, as well in the medical field, where surgical components and implants are produced with EDM [3].

EDM is a multi-parameter process where complex underlying physical mechanisms are taking place. The majority of studies on EDM focus on the parameters that affect machining performance most, namely, the machining voltage, the pulse-on current (Ip), the pulse-on time (Ton), and the duty factor (DF). It is necessary to differentiate between open-circuit (Vo) and close-circuit (Vc) voltages when discussing machining voltage, as they have different effects on the process [4,5]. The performance indexes that are usually employed to study EDM are the Material Removal Rate (MRR), the Tool Material Removal Rate (TMRR), and the Tool Wear Ratio (TWR), and regarding the Surface Quality (SQ), the Surface Roughness (SR) in terms of the mean surface roughness (Ra) and the maximum peak to valley height (Rt). Moreover, since EDM is a thermoelectric process, metallurgical transformations are taking place; hence, a layer of amorphous material is formed on the machined surface, referred to as a White Layer (WL), while beneath it, a Heat Affected Zone (HAZ) is also developed. These indexes mentioned above are directly linked to the machining parameters, and thus, extensive research has been conducted to better understand and for subsequent optimization [5,6]. In EDM, different materials, even different alloys belonging to the same alloy class, have dissimilar behavior. Hence, extensive research and investigation are required for the machining of each particular alloy, using a specific electrode material and for a certain range of process parameters. Steel alloys, which find wide use in many modern industrial applications, gather intense research and scientific interest concerning their machining with EDM [7].

In a milestone study of Lim et al. [8] the machining of different tool steels with EDM was investigated, and specifically the solidification microstructures of the different tool steels. This research is a perfect example of the unalike behavior of steels during EDM, indicating the necessity of their extended and comprehensive study. Che Haron et al. [9] presented a comparative study for machining XW42 tool steel using copper and graphite electrode, concluding that the copper electrode is suitable for the roughing process, while the graphite electrode is suitable for the finishing process. The work of Younis et al. [10] mainly focused on the effect of the electrode material on the machined surface quality. Two different electrode materials and workpiece materials were tested, namely Dura graphite 11 and Poco graphite EDMC-3 and DIN 1.2080 and DIN 1.2379 steels, respectively. The authors deduced that the SQ is strongly affected by both the machining parameters and the employed electrode material. Sharif et al. [11] conducted experiments concerning the machining of 316 L stainless steel with EDM. Among the other conclusions, authors inferred that pulse-on current, pulse-on time and pulse-off time are significant factors, while, according to the Response Surface Method (RSM) that was utilized for statistical analysis of the results, the servo voltage does not have a significant effect. In the work of Barenji et al. [12] the MRR and the TWR in the machining of AISI D6 tool steel with EDM was studied. The control parameters were the pulse-on time and current and the open-circuit voltage, while, by applying RSM, they presented the modeling and optimization of the process. Another comparative research was conducted by Mishra and Routara [13] regarding the differences in machining a typical and a hardened EN-31 steel with EDM. The control parameters were the pulse-on current and time, the duty factor, and the gap voltage, while the machining performances were estimated in terms of the MRR and TWR. The enhanced hardness resulted reduced the MRR and increased tool wear. The effects of process parameters on the performance of electrical discharge machining of AISI M42 was studied by Choudhary and Singh [14]. The control parameters were again the pulse-on current, time, and voltage, utilizing a Taguchi Design of Experiment (DOE), while additionally, they conducted experiments with reverse electrode polarity. The machining of H13 die steel using different electrode materials and for different pulse-on current and time was experimentally studied by Bahgat et al. [15], while in the work of Rani et al. [16], the improvement of metal removal efficiency through executing the amendment of electrical circuits in EDM for machining Eglin steel was discussed. The machining of Eglin steel with EDM was also studied by Sahayaraj et al. [17] employing an Artificial Neural Network (ANN) model to predict the machining process results. The study of Dinesh et al. [18] pertains to the machining of oil hardened non-shrinking die steel with powder mixed EDM. The study was focused on the impact of pulse-on time, duty factor, and powder concentration, while the effect of pulse-on current was not included in the mentioned study. The machining of steels with powder-mixed EDM was also studied in the recent work of Huu [19] and Jeavudeen et al. [20], where titanium powder and alumina powder was utilized, respectively. Finally, the machining of AISI H13 steel with EDM has been studied by Gopal et al. [21] and Mohanty et al. [22]. The effect of the machining parameters on the process was investigated, while the work of Gopal et al. indicated how the pre-process of the electrode’s material may significantly impact the machining results. From the above brief literature review, the scientific interest and practical importance of the research regarding the machining of steel alloys with EDM can easily be deduced, especially for alloys with a wide commercial utilization [23].

The tool steel with the commercial name CALMAX (1.2358) is a chromium-molybdenum-vanadium alloy steel, of high toughness and ductility, good wear resistance and stability in hardening, and good weldability. It is suitable for both cold work and plastic applications, namely, it is typically used for injection molds for the production of plastics, tools for cutting and machining plastics, dies, scissors, cutting blades, punches, tools with complex shapes for cold forming, and inserts.

The current study pertains to the machining of CALMAX steel with EDM. The main aim is to present a comprehensive analysis of how the major machining parameters, i.e., the pulse-on current and time and the open-circuit voltage affect the process and its results. More specifically, a Taguchi DOE has been adopted with the control parameters being the pulse-on current, the pulse-on time, and the open-circuit voltage. The machining performances were estimated in terms of the MRR, TMRR, and TWR, while the SQ was studied by measuring the SR (Ra, Rt, Rsk, and Rku) and the Average White Layer Thickness (AWLT). Furthermore, the machined surfaces and the corresponding cross-sections were observed under optical and SEM microscopy, while through EDX maps, the materials diffusion and the after-process elements distribution were defined. Moreover, the changes in the micro-hardness of the WL and the HAZ were estimated through micro-hardness measurements. Finally, and after the necessary Analysis of Variance (ANOVA), based on grey relational analysis, multiparameter optimization was performed, determining a different optimal set of parameters according to different performance criteria. The current study, by covering a wide range of machining conditions, aims to constitute a reference for further research, as well a guideline for more practical industrial applications, since, to the knowledge of the authors, no systematic research of CALMAX with EDM can be found in the relevant international literature.

## 2. Materials and Methods

**Figure 1:**
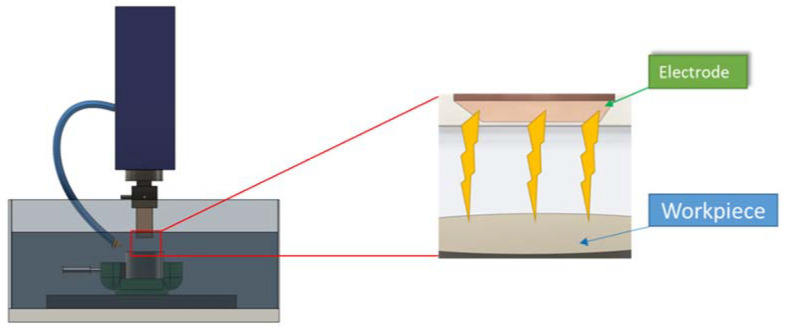
Graphical representation of the EDM process.

The experiments were carried out on an Agie-Charmilles Roboform 350 Sp industrial-type die-sinking EDM machine. As Figure 1 shows, a rectangle copper electrode with nominal dimensions of 14 × 20 mm was employed, while the workpieces were rectangle slices of CALMAX steel. The utilized dielectric fluid was a synthetic hydrocarbon oil (kerosene), which was properly channeled into the working tank through a flushing nozzle. In Table 1, the CALMAX typical chemical composition is listed, along with its main thermophysical properties. In the current series of experiments, rectangle voltage pulses were employed, with control parameters the pulse-on current, the pulse-on time and the open-circuit voltage. Aiming to cover a wide range of machining conditions, i.e., machining power and per-pulse energy, each control parameter had 4 levels, while the rest of machining conditions were kept constant. Namely, the duty factor was set at 50%, the close circuit voltage 30 V, the flushing pressure at 0.7 MPa, and the nominal depth of cut at 0.5 mm. The combinations of the machining conditions were specified based on the Taguchi DOE method (see the following sub-section). The experimental parameters are presented in Table 2.

The MRR, TMRR, and TWR were calculated according to Equations (1)–(3), respectively:

(1) $$\mathrm{MRR}=\frac{W_{st}-W_{fin}}{t_{mach}}\cdot \frac{1}{\rho_{w}}$$

(2) $$\mathrm{TMRR}=\frac{E_{st}-E_{fin}}{t_{mach}}\cdot \frac{1}{\rho_{el}}$$

(3) $$\mathrm{TWR}=\frac{El_{st}-El_{fin}}{W_{st}-W_{fin}}$$

with MRR in mm^3^/min, TMRR in mm^3^/min, TWR in gr/gr, ρ_w_ and *ρ_el_* the workpiece and electrode material density, respectively in gr/mm^3^, *t_mach_* the machining time in min, *W_st_* and *W_fin_* the workpiece weight before and after the machining in gr, while *El_st_* and *El_fin_* are the electrode’s weight before and after the machining, respectively, in gr. It has to be pointed out that in-between the experiments any depositions from the electrode’s working surface were removed through grinding, in order for the experimental conditions to remain unchanged. The SR, i.e., Ra, Rz, RSk, and Rku, were measured using a Keyence VHX-7000 optical microscope equipped with specialized lenses and by utilizing the focus variation method. The measurements were conducted following the ISO 25178-2 standards, and based on the obtained SR values, the cut off length was defined at 0.8 mm. Since EDM is a chaotic process in micro-scale, it results in a uniform and isotropic SR, without any particular periodic variation of the roughness in respect of a specific orientation. Hence, Ra, Rt, and RSk can be considered as representative and proper SR indexes to describe and evaluate the roughness of a surface that was machined with EDM.

**Figure 2:**
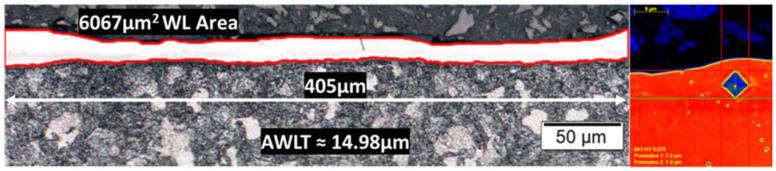
Snapshots from the measuring of the AWLT and the WL micro-hardness.

Finally, the machined surfaces cross-sections were polished and properly chemically etched, in order for the microstructural differences of the WL to be identified and its mean thickness to be measured. The AWLT is calculated as the quotient of the respective area to the corresponding length. Furthermore, using the EDX maps, the possibility of material diffusion in the WL and the HAZ was considered, while the changes in WL and HAZ hardness were estimated throughout micro-hardness measurements. The micro-hardness measurements were carried out on a Struers DuraScan-70 tester, employing the Vickers micro-hardness test and applying 25 gr load (HV0.025). Recurring micro-hardness measurements in the bulk material and the WL were made, and the conclusions pertain to their average values; Figure 2 depicts the measurement process of the AWLT and the micro-hardness.

### 2.1. The Taguchi DOE Method

Apart from the simplest full factorial DOE, where all the possible factors combinations are tested, there are effective statistical and DOE methods, from which, although a lower number of experiments are conducted, reliable information can still be deduced. The orthogonal arrays approach, which is employed by the Taguchi DOE method, is one of them. The term orthogonal means that the columns of the arrays are balanced. Balanced, in turn, means at first that within each column there are an equal number of levels, as well that the combinations of levels between any two columns are also equal in number [24]. In the current study, three control parameters were considered, each one having four levels. By utilizing the Taguchi DOE method, the necessary number of experiments was limited from 64 (4^3^) to 16, based on the L_16_ orthogonal array. The control parameter combinations are listed in Table 3.

### 2.2. Grey Relational Analysis

Grey Relational Analysis (GRA) is widely employed for analyzing the relations between discrete data sets and for decision making in multi-attribute systems. Because GRA is a straightforward method, based on original data and with easy-to-make calculations, it has been established as one of the most adopted methods in the relevant field. The method proposes a dependence estimation in order to measure the correlation degree between the control factors [25].

The procedure can be summarized in the following main steps:

First, all alternatives have to be transformed into a comparable sequence, a step that is called grey relational generating. The process is analogous to normalization, and in systems with *m* alternatives and *n* attributes, the *i^th^* alternative can be expressed as *Y_i_ = (y_i1_, y_i2_, …, y_ij_, y_in_)* with *y_ij_* the performance value of attribute *j* of alternative *i*. The term *Y_i_* is translated into the comparability sequence *X_i_ = (x_i1_, x_i2_, …, x_ij_, x_in_)* according to Equations (4) and (5), for larger-the-better attributes:

(4) $$x_{ij}=\frac{y_{ij}-min\left(y_{ij}\;for\;i=1,2\ldots ,\;\;m\right)}{max\left(y_{ij}\;for\;i=1,2\ldots ,\;\;m\right)-min\left(y_{ij}\;for\;i=1,2\ldots ,\;\;m\right)}\;for\;\begin{array}{c} i=1,2,\ldots ,\;m\\ j=1,2,\;\ldots ,\;n \end{array}\;$$

(5) $$x_{ij}=\frac{max\left(y_{ij}\;for\;i=1,2\ldots ,\;\;m\right)-y_{ij}}{max\left(y_{ij}\;for\;i=1,2\ldots ,\;\;m\right)-min\left(y_{ij}\;for\;i=1,2\ldots ,\;\;m\right)}\;for\;\begin{array}{c} i=1,2,\ldots ,\;m\\ j=1,2,\;\ldots ,\;n \end{array}$$

After the grey relational generating process, all the performance values are scaled between 0 and 1. For an attribute *j* of alternative *i*, values *x_ij_* equal 1, which means that the performance of alternative *i* is the best one for attribute *j*. Conceptually, an alternative would be optimal if all of its performance values are equal to 1, simultaneously. Nevertheless, in real systems, this ideal alternative rarely exists; thus, an alternative with a comparability sequence close to the reference has to be identified. To determine how close a *x_ij_* value is to the optimal value *x*_0*j*_ for attribute *j*, the grey relational coefficient *γ(x_0j_,x_ij_*) has to be calculated according to Equation (6):

(6) $$\gamma \left(x_{0j},x_{ij}\right)=\frac{\Delta_{min}+\zeta \Delta_{max}}{\Delta_{ij}+\zeta \Delta_{max}}\;\;for\;\begin{array}{c} i=1,2,\ldots ,\;m\\ j=1,2,\;\ldots ,\;n \end{array}\;\;\;\;\;\;\;with\;\begin{array}{c} \begin{array}{c} \Delta_{ij}=\left|x_{0j}-x_{ij}\right|\\ \Delta_{min}=min\left(\Delta_{ij}\right) \end{array}\\ \Delta_{max}=max\left(\Delta_{ij}\right)\\ \zeta =0.5\;the\;distinguished\;coefficient\; \end{array}$$

Finally, after the calculation of the entire grey relational coefficient *γ*(*x_0j_, x_ij_*), the grey relational grade is estimated using Equation (7):

(7) $$\Gamma \left({Χ}_{0},{Χ}_{i}\right)=\overset{n}{\underset{j=1}{\sum }}w_{j}\gamma \left(x_{0j},x_{ij}\right)\;\;\;for\;\;\;i=1,2,\ldots ,\;m$$

with *Γ*(*Χ_0_, Χ_i_*) the grey relational grade between *X_i_* and *X*_0_, which pertains to the correlation level between the reference optimal sequence and the comparability sequence. w_j_ is the weight of attribute *j*, which depends on the inherent structure of the problem/system, with $\sum_{j=1}^{n}w_{j}=1$.

In the current study, the optimization was proposed in respect to the four major machining performance indexes, namely the MRR, the TWR, the Ra, and the AWLT. The MRR and the TWR are directly related to the efficiency and economic feasibility of the process, while the Ra and the AWLT are related to the part’s quality. Considering that the aim is the maximization of the MRR with the simultaneous minimization of the TWR, Ra, and AWLT, Equations (4) and (5) were employed, respectively.

## 3. Results and Discussion

**Figure 3:**
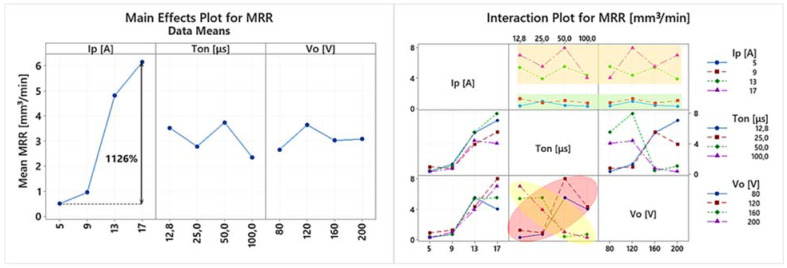
Main effects plot and the interaction plot for MRR.

**Figure 4:**
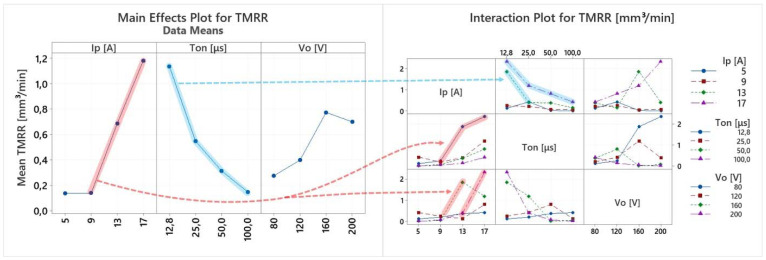
Main effects plot and the interaction plot for TMRR.

**Figure 5:**
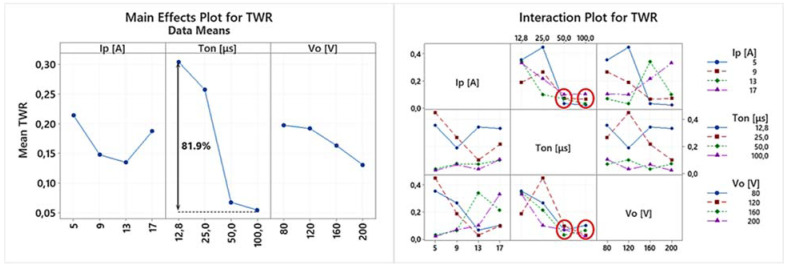
Main effects plot and the interaction plot for TWR.

**Figure 6:**
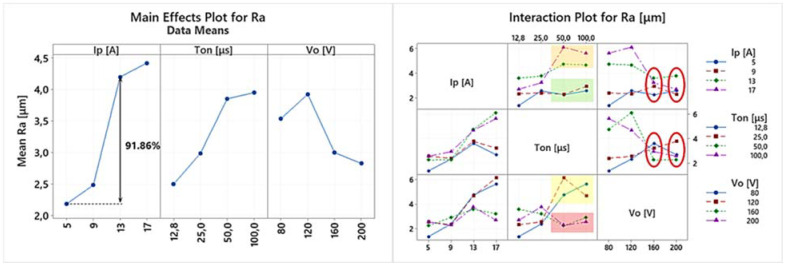
Main effects plot and the interaction plot for Ra.

**Figure 7:**
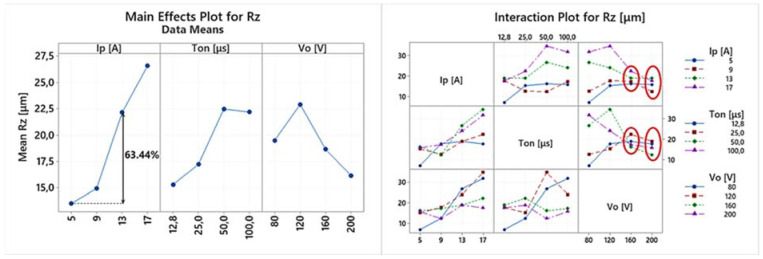
Main effects plot and the interaction plot for Rz.

**Figure 8:**
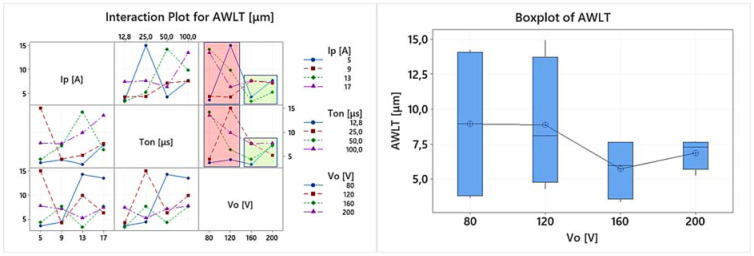
Interaction plot for AWLT and the boxplot diagram for AWLT in respect of the V_o._

**Figure 9:**
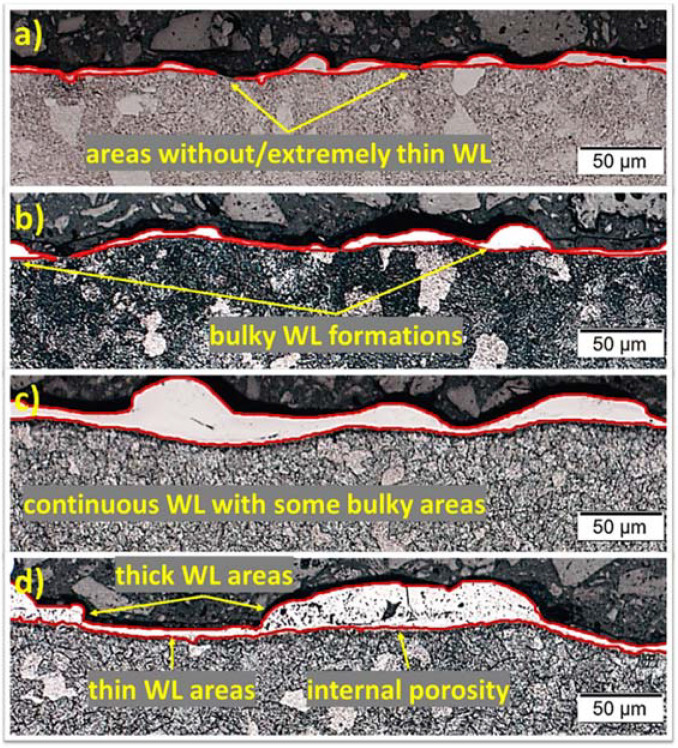
Machined surfaces cross-sections for pulse-on current, pulse-on time, and open-circuit voltage. (**a**) 5 A–12.8 μs–80 V, (**b**) 9 A–25 μs–80 V, (**c**) 13 A–50 μs–80 V, and (**d**) 17 A–100 μs–80 V.

EDM is a multi-parameter process, where complex physical phenomena are taking place, resulting in a nonlinear response of the system. Hence, any conclusion has to be carefully deduced, avoiding superficial analysis, by highlighting the importance of the in-between control parameter interactions, and how they actually affect the machining results. Thus, the following ANOVA will be based on both the main effects plots as well the interaction plots of the machining performance indexes. In Table 4 the experimental results are presented, based on which the main effects plot and the interaction plot of Figure 3, Figure 4, Figure 5, Figure 6, Figure 7, Figure 8 and Figure 9 emerged.

### 3.1. Material Removal Rate, Tool Material Removal Rate, and Tool Wear Ratio

ΜRR, TMRR, and TWR are three performance indexes of major significance which are directly related to the efficiency and economic feasibility of the process. They are strongly affected by the machining parameters (i.e., Ip, Ton, and Vo) and more specifically by the machining power and the per-pulse energy. Nevertheless, the intuitive hypothesis that higher machining power and/or per-pulse energy will automatically lead to a higher MRR is incorrect, mainly because of three underlying mechanisms: the plasma channel growth, the debris concentration in-between the electrode and the workpiece, and the carbon decomposition. More specifically, as the plasma channel expands (e.g., for higher pulse-on times) it consumes a significant amount of energy, while at the same time, the energy density is decreased [1,3,26]. Moreover, the increase of the MRR results in a higher debris concentration in-between the electrode and the workpiece, a concentration that impacts the flushing efficiency. Remaining debris in the gap between the electrode and the workpiece not only consumes energy as it re-melts, but also may destabilize the process and/or cause arcing conditions [27]. Finally, the carbon from the dielectric is decomposed and bonded on the electrode, forming a “shield layer”, which at the same time acts protectively for the tool electrode, limiting its wear, and is unbeneficial for the MRR [6]. The brief aforementioned theoretical analysis presupposes and explains any peculiar behavior of the performance indexes.

In Figure 3 the main effects plot and the interaction plot for the MRR are presented. Based on the main effects plot, it can be deduced that the Ip has a clear impact on the MRR, with its increase leading to a higher MRR. Specifically, the mean MRR increased by 1126% as the pulse-on current increased from 5 to 17 A. On the contrary, the Ton and the Vo seem to have a minor and vague effect on the MRR. By perusing the interaction plot, a clearer view of the process can be gained. The Ip has indeed a clear impact on the MRR for almost all the pulse-on currents and open-circuit voltages. They follow the same upward trend up to 13 A, and only for 17 A there is a deviation between different Tons and Vos. At the same time, it is noticed that for low pulse-on currents, i.e., 5 and 9 A (see the green area), there is almost no difference in MRR as the Ton and Vo change, while the high pulse-on currents, i.e., 13 and 17 A (see orange area), are sensitive to changes of the Ton and Vo. This differentiation between low and high values also incurs for the Vo, where the 80 and 120 V have a different behavior in contrast to the 160 and 200 V; see the red and yellow areas, respectively. Hence, it can be reasonably deduced that the combination of the machining parameters is of major importance and not the parameters by themselves, while additionally, the system has a different response regarding low and high machining powers and per-pulse energies.

A direct comparison between different mold steel is difficult and tricky, since each alloy holds some unique properties and behavior. Nevertheless, and in light of some general assessment for machining CALMAX with EDM, a careful and targeted comparison is quoted. The main conditions for a scientifically correct comparison are for the material to have some similarities with the studied material (e.g., content of the main alloying element or the main use), the machining conditions to be similar, and the data to be up to date, avoiding some misleading conclusions based on outdated studies. Keeping that it mind, in the study of Valaki and Rathod [28] we find that for M238 HH grade, a cold work plastic mold steel, the voltage and pulse-on time indeed have less effect on the MRR compared with the pulse-on time. Moreover, generally, a higher MRR was achieved, a result that can be attributed to the different behavior of the material, as well the utilization of a lower open-circuit voltage. In the aforementioned study, lower open-circuit voltages were used (up to 80 V), and based on the obtained results, and as a rule of thumb, it is deduced that low voltages lead generally to a higher MRR. These conclusions are also supported by the work of Aich and Banejee [29] regarding the machining of M2 grade with EDM, where higher MRRs were measured although lower open-circuit voltages were used, compared with our study.

In Figure 4 the main effects plot and the interaction plot for the TMRR are presented. For the main effect plot it could be deduced that increase in the pulse-on current results in a higher TMRR, while increase in the Ton leads to a lower TMRR. Nevertheless, this conclusion would be inaccurate, hence, a more in-depth analysis is necessary. More specifically, although the increase of Ip generally results in a slightly higher TMRR, the great increase is mainly attributed only to certain machining conditions, i.e., 12.8 μs pulse-on time and/or 160 and 200 V open-circuit voltage; see the red line. On the contrary, for the rest of the machining conditions, the increase of Ip only minorly impacted the TMRR. Similarly, the decrease of the TMRR for higher pulse-on times is mainly due to the significant decrease for 13 and 17 A—see the blue line—while for 5 and 9 A, the TMRR remained almost constant in respect of the Ton. Considering the aforementioned analysis, any quantitative estimation based on the mean values of the main effects plot for the TMRR was avoided, as it could be misleading, while it is deduced that the TMRR is significantly increased for specific machining combinations, a fact that must always be taken into account during machining planning, since it is related with the process’ efficiency and its economic viability.

Closing the current sub-section, the main effects plot and the interaction plot for the TWR are presented in Figure 5. It has to be pointed out that the TWR constitutes the percentage comparison of the electrode and workpiece wear, which emerges as a result of the superposition of the MRR and TMRR; thus, it is expected that the TWR will differ from both of them. Indeed, according to Figure 6, it can be concluded that the Ip has an ambiguous impact on the TWR, while the Ton seems to affect it in a more predictable way. As the pulse-on time increased from 12.8 to 100 μs, the mean TWR decreased about 81.9%, a fact that is not surprising and is in line with the graphite shielding mechanism that was aforedescribed. At the same time the increase of the Ip results in either an increase or decrease of the TWR depending on the combination of the Ton and Vo. The vague impact of pulse-on current on the TWR is also observed in machining of M238 HH grade with EDM, while the most significant parameter for the TWR is again the pulse-on time [28].

Finally, two interesting observations have to be made; first, for pulse-on times 50 and 100 μs, almost the same TWR was measured, regardless of the utilized pulse-on current or the open-circuit voltage, and secondly, different machining combinations may result in almost the same MRR but entirely different TWRs, e.g., experiments 9 and 11 that both have an MRR of approximately 5.5 mm^3^/min but their TWRs are 0.342 and 0.068, respectively. Again, the significance of selecting the optimal process parameters during machining planning is suggested.

### 3.2. Surface Roughness, Average White Layer Thickness, and Heat Affected Zone

The surface roughness and the surface quality are substantial parameters of the process and machining planning, since the manufactured components have to meet strict quality standards [30]. Moreover, in cases where a post-process is needed, the surface and subsurface characteristics have to be well known and accurately defined in order for the subsequent treatment to be suitably planned. Hence, the machined surfaces after EDM have to be extensively studied, not only due to academic and scientific interest, but for practical reasons as well.

Conceptually, in EDM, each spark occurring melts or ablates an amount of material, leaving behind a tiny crater. Just as the total material removal is a result of millions of successive sparks, so the SR is the accumulative result of these tiny craters and their superposition. It can be easily deduced that the SR and the SQ are directly related with the machining parameters; nevertheless, the process’ stochastic nature and its chaotic behavior in micro-scale does not allow a strictly deterministic interpretation and approach [31]. Based on the literature, the craters’ morphological characteristics are impacted by the machining power and the per-pulse energy, namely, the pulse-on current mainly affects the craters’ depth, while the pulse-on time mainly affects their width [1]. At the same time, only an amount of the molten material is removed by the workpiece surface, while the rest is re-solidified. Moreover, ablated material that has not been efficiently flushed away may cool down rapidly and re-adhere on the surface, forming “debris adheres”. These re-solidified and re-condensed layers of material, well known as white layer, are amorphous and have distinctive properties in comparison to the bulk material. The WL thickness and its morphological characteristics mainly depend on the machining parameters, i.e., Ip, Ton, and Vo, the electrode and workpiece material, and the utilized dielectric fluid. Typical formations of the WL are crater marks, uneven depositions of melted and re-solidified material in the form of islets, scattered debris, inclusions, pockmarks, and cracks. Cracks are developed due to the combined effect of residual and thermal stresses, while the topical high gradients in pressure and temperature favor their initiation and further development [1,32].

In Figure 6, the main effects plot and the interaction plot for Ra are presented. From the main effects plot it can be deduced that higher pulse-on current results in a higher mean Ra. More specifically, the mean Ra increased by 91.86% as the Ip increased from 5 to 13 A. From the apposition of main effects plot and the interaction plot is emerged that, up to 13 A, for all the machining parameters combinations, the Ra values seemed to follow a common trend, while for 17 A, there is a significant deviation between the low and the high values of Ton and Vo. This deviation led to the limitation of the quantitative comparison between 5 and 13 A, instead of 5 and 17 A. This significant differentiation between low and high values can also be observed in the interaction plot of pulse-on time, where the lower values of Ip and V_o_ (orange and yellow areas, respectively) clearly separate from their corresponding higher values (green and red areas, respectively). Finally, an interesting conclusion that can be deduced from the interaction plot of V_o_ is that the Ra values for the low open-circuit voltages have a considerable deviation depending on the machining parameters combination, while for the higher Vos (160 and 200 V), the Ra values tend to converge, with Ra becoming less sensitive to the effect of Ip and T_on_.

In Figure 7 the main effects plot and the interaction plot for Rz are presented. It is reasonable that Rz follows similar trends with Ra; nevertheless, Rz, due to its definition, is a more sensitive and mutable index, since a random debris/material deposition may result in its increase. From the main effects plot, it can be concluded that a higher pulse-on current results in a higher mean Rz, namely, there is a 63.44% increase of the mean Rz between 5 and 13 A. Again, for 17 A, a significant deviation in Rz values is observed; thus, 17 A is not included into the previous comparison since the mean Rz value for 17 A cannot be considered as representative. Considerable deviation in Rz values depending on the machining parameters combination is also present for the high values of the pulse-on time, as well as for the lower values of the open-circuit voltage. Like in Ra, only for the high open-circuit voltages, i.e., 160 and 200 V, the Rz is stabilized, converged, and becomes less sensitive to the change of Ip and Ton. As an overall and brief conclusion regarding the Ra and Rz, it can be said that the SR is strongly affected by the combinations of the machining parameters, rendered, by case, more or less sensitive in their change. Hence, during the machining planning, the SR always has to be considered as an important criterion, by aiming not only for the higher MRR and/or lower TWR, but at the same time, for the desired SQ and SR specifications [30].

Concerning the surface skewness (Rsk), some interesting results emerged. A negative value of Rsk means a surface that is mainly made up of valleys, whereas a surface with a positive skewness mainly contains peaks and asperities. The Rsk values range between −0.52 and 1.07, i.e., mostly close to zero, indicating that the height distribution of the machined surface is, more or less, symmetrical around the mean plane. This is actually an interesting conclusion, since it can be attributed to the craters’ formation mechanism (it will be discussed later in the study) where a crater is formed by the material removal; nevertheless, material is also ejected forming its rims. Hence, the surface not only consists of successive overlapped cavities, but of bulky flanks and ridges as well, which results in this skewness. At the same time, the kurtosis index (Rku), which describes the sharpness of the profile, takes values around 3 (2.59 < Rku < 4.78), suggesting that the machined surfaces are not compulsorily platykurtic (Rku < 3) or leptokurtic (Rku > 3). Thus, as an overall conclusion can be deduced that the sharpness and the skewness depend on the machining parameters combination and the balance between the material removal (i.e., crater formation) and the material’s insufficient flushing with the simultaneous formation of bulky areas and rims [33].

The WL strongly affects the machined SR, while it concurrently impacts the machined surface properties. Hence, the measuring and definition of the AWLT is of extreme interest and importance, as well the determination of the WL’s morphological characteristics. The interaction plot and the boxplot diagram for the AWLT are presented in Figure 7. The correlation between the AWLT and the Ra and Rz is obvious, since it is easily found that the AWLT takes its lower values for the higher open-circuit voltages, i.e., 160 and 200 V. Moreover, based on the interaction plot and the boxplot diagrams, two different machining areas can be distinguished, one with high deviation of the AWLT depending on the combination of the machining parameters (red area—low open-circuit voltages) and one with low deviation of the AWLT values, where the combination of the machining parameters have minor impact on the AWLT (green area—high open-circuit voltages). Summarizing, for the higher Vos, a lower and less different AWLT were measured, a fact that can be explained by interpreting the physical meaning and effect of the open-circuit voltage. A higher Vo allows for a wider gap between the electrode and the workpiece, since the dielectric fluid electrical constant will break at a greater distance between the working electrode and the workpiece. Thus, a more efficient flushing is taking place, resulting in a higher portion of the molten material being removed from the workpiece surface, leaving behind less material volume to form a thinner WL. Hence, in cases where surfaces free of WL are needed, higher open-circuit voltages should be used, or for lower open-circuit voltages, a mindful selection of the machining parameters combination has to be made, using, for example, a low pulse-on time by bearing in mind that for Ton 12.8 μs a low AWLT has been measured.

**Figure 10:**
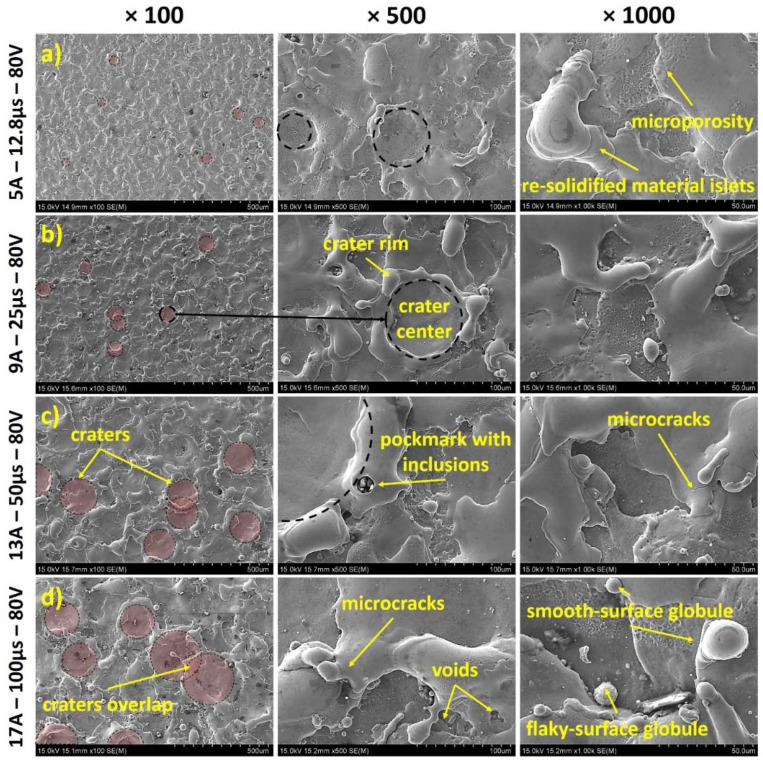
Machined surfaces SEM images for pulse-on current, pulse-on time, and open-circuit voltage. (**a**) 5 A–12.8 μs–80 V, (**b**) 9 A–25 μs–80 V, (**c**) 13 A–50 μs–80 V and (**d**) 17 A–100 μs–80 V.

Along with the measuring of the AWLT, a comprehensive study of the machined surface has to include an analysis of the WL morphological characteristics. Thus, in Figure 9 and Figure 10 the surfaces’ cross-sections and SEM images of the machined surfaces are depicted, respectively. Since for higher open-circuit voltages, a thin WL is formed and is less sensitive to changes of the machining parameters, it was considered reasonable to focus the investigation and presentation on the lowest Vo, i.e., 80 V, and for different pulse-on current and time combinations. For 5 A and 12.8 μs (Figure 9a), a very thin and discontinuous WL has been formed, while there are some more bulky spots. For 9 A and 25 μs (Figure 9b), the WL is still very thin, but now, it is almost continuous, and the bulky spots gradually grow in volume. For higher per-pulse energy, i.e., 13 A and 50 μs (Figure 9c), a continuous and with higher average thickness WL has been formed. Although some degree of uniformity can still be observed, thinner and bulky areas follow one another. For even higher per-pulse energy, i.e., 17 A and 100 μs (Figure 9d), the WL has entirely lost its uniformity, and now, it is clearly divided in thin and thick areas. Moreover, in the thicker areas, macro and micro inner porosity, voids, and cracks appear. Obviously, the morphological characteristics of the WL strongly depend on the machining parameters, while the major impact and the close correlation of the WL with the SR is verified.

To fully understand the WL formation mechanisms, SEM images of the machined surfaces should be studied in juxtaposition with the respective cross-sections. Thus, in Figure 10, SEM images of the machined surfaces for the same parameter combinations as those of Figure 9 are depicted. The surfaces are scattered with craters, which were formed by the sparks and the plasma channels. As the per-pulse energy is increased, craters become larger and, hence, more easily identified, while the final surface morphology is the accumulative result of overlap of the craters. The craters’ formation mechanism is a very interesting topic that has been addressed in the study of Nowicki et al. [34]. In the craters, two discrete areas can be observed, the crater’s center area and the crater’s rim. The center area is smooth, since the molten material was uniformly ejected due to the high temperature and pressure at the center of the plasma channel [35]. Part of the molten material that was not efficiently flushed away, forms the crater’s rim, with these elevated crater wall and flanks being the bulky formations that were observed in the surfaces’ cross-sections. For more intense machining parameters, molten material is ejected farther, the crater’s rim becomes voluminous and more irregular, composed by many layers due to the interaction of neighboring craters. Thus, the surface mainly consists of the craters’ smooth central areas and islets of layered re-solidified material, with voids, pockmarks, and inclusions as result of the extremely intense temperature and pressure conditions that take place topically. When the pockmarks become too fine, they appear in the form of microporosity, a characteristic that may affect the surface’s corrosion resistance. On the other hand, the surface is almost free of macrocracks, and only a few microcracks can be observed, a significantly beneficial feature regarding the corrosion resistance. The formation of micro- and macrocracks is a typical characteristic of the EDMed surfaces [7,36]; thus, the absence or the very limited formation of such cracks definitely confers a significant property of the current alloy, at least in the current range of machining conditions. Finally, especially for the higher per-pulse energies, globule formations of re-solidified material have been re-attached on the surface. The size and the texture of these globules depend on their formation mechanism. Globules that have been formed due to incomplete evaporation tend to be larger and with smooth surfaces, in contrast with those that emerged from re-condensed vapor material, which are smaller and with flaky surfaces [37]. Based on the above analysis, the successive thin and thick WL areas of the cross-section can be explained, along with the “formation history” of the surface roughness and texture. 

**Figure 11:**
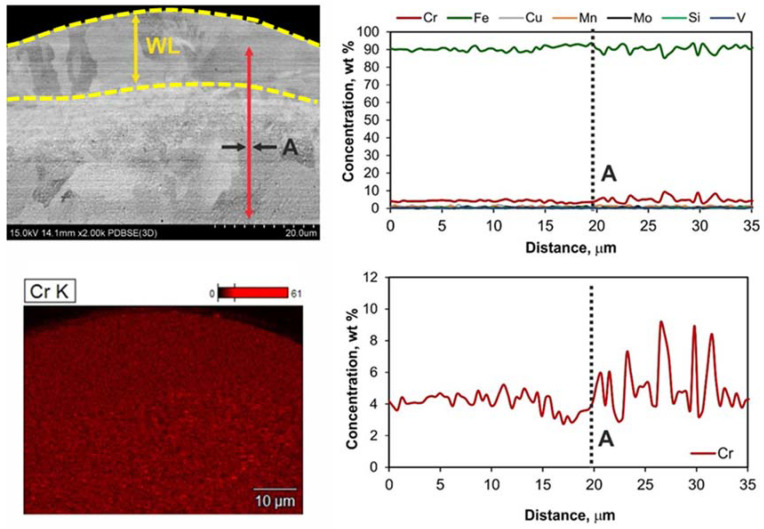
Representative results from EDX line analysis for 17 A, 100 μs, and 80 V.

**Figure 12:**
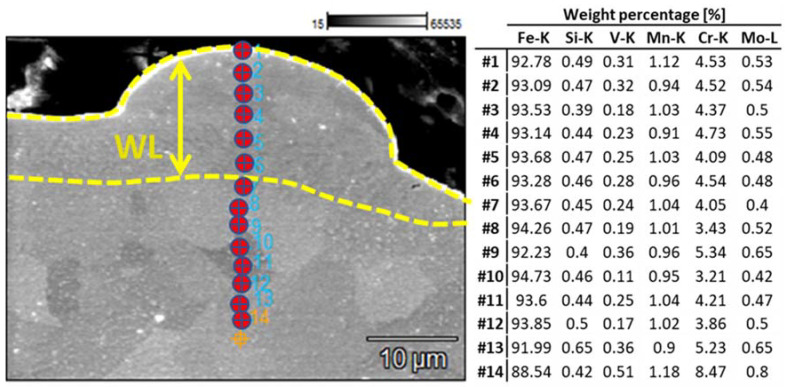
EDX point analysis for 13 A, 50 μs, and 80 V.

From the conducted micro-hardness measurements, it is deduced that the WL micro-hardness has significantly increased compared to the bulk material. More specifically, the bulk’s material micro-hardness was 200 HV_0.025_, while the micro-hardness of the WL ranges from 800 HV_0.025_ up to 890 HV_0.025_, an increase of over 400%. The deviation of the WL micro-hardness can be attributed to its inherent characteristics, such as inclusions, microporosity, and micro-voids; nevertheless, the increase is notable and undoubted. These results are in line with bibliographic references regarding the micro-hardness of the WL in stainless and work steel [38,39]. It is worth mentioning that the increased micro-hardness does not occur due to different chemical compositions but only as the result of the material’s amorphization. Figure 11 shows the results representative of the chemical composition analysis (Cr concentration map, linear analysis of the concentration of steel elements, and the electrode) for the 17 A, 100 μs, and 80 V. The presented results prove that, as a result of the interaction of the electrode with the steel surface, probably the dissolution of chromium carbides, which dissolve in steel first, took place; therefore, the steel matrix was enriched with chromium. The content of elements included in the tested steel mainly remains unchanged, as it is deduced by the EDX analysis of Figure 12. This conclusion is in agreement with the study of Ibrahim et al. [40], where it was deduced that the enhanced hardness value in Fe-based metallic glasses is directly proportional to the amorphous phase content percentage. The increased micro-hardness of the EDMed surfaces has always to be taken into consideration, since it majorly affects the surface’s tribological characteristics, especially if the machined component is part of a mechanism.

### 3.3. Optimization Based on Grey Relational Analysis

As it has already been mentioned, the optimization of EDM is not straight forward, since, during the machining, competitive physical mechanisms are taking place. Thus, the optimal parameters combination for the highest MRR do not necessarily coincide/agree with that for the lowest TWR or the lowest Ra, and hence, a multi-objective parametric optimization method has to be adopted. In the current approach, GRA has been employed for the estimation of the optimal machining parameters according to two different criteria sets. In the first case, the optimization pertains to the maximum MRR and the lowest TWR, while in the second one, it considers the maximum MRR, the lowest TWR, and the lowest Ra as well. Based on Equations (4) and (5), for the MRR and TWR/Ra, respectively, the grey relational coefficients and the corresponding Grey Relational Grades (GRG) have been calculated (see Table 5).

Based on the GRG, for both optimization scenarios, the optimal machining parameters combination is 17 A, 50 μs, and 120 V (pulse-on current, pulse-on time, and open-circuit voltage, respectively). At this point, an important remark has to be made; the GRG, when only the MRR and the TWR were considered as optimization indexes, is significantly higher (20%) than the GRG when the Ra was also included as the 3rd objective. Thus, it is clearly deduced that when more optimization objectives are taken into account, it becomes more and more difficult for a general optimal to be accurately defined. At the same time, the optimization process becomes even more vague and uncertain, since alternative combinations with almost the same GRG may arise. For example, the GRG for 5 A, 50 μs, and 160 V is almost equal with the aforementioned optimal GRG: 0.673–0.688, an approximately 2% difference. Hence, in optimization, only the by case absolutely necessary performance indexes should be taken into consideration. When the minimization of the ALWT was included in the RSM analysis, a different machining parameters combination emerged as optimal, i.e., 5 A, 50 μs, and 200 V. Observing the GRC, it is deduced that for this specific combination, the GRCs of the TWR, Ra, and AWLT are significantly high, while the GRC of the MRR is low. Since MRR represents only one of the four optimization indexes, the three other outputs become dominant and a low MRR GRC is overshadowed. A more practical interpretation of the GRA results indicates that when only the MRR and TWR are considered, meaning the major parameters are the productivity and the efficiency of the process, the GRG was dominated by the MRR and was also very high in an unambiguous way. When the Ra was also taken into account along with the MRR and TWR, the GRG decreased and the process moved to a more vague optimization area. Finally, when the AWLT was also included, and now there are two parameters that are related with the SQ (i.e., Ra and AWLT), the GRG increased again and the MRR no longer significantly affected the optimization process.

## 4. Conclusions

In the current paper, an experimental study regarding the machining of CALMAX, a chromium-molybdenum-vanadium tool steel, with EDM was presented. An orthogonal Taguchi DOE was adopted, with control parameters pulse-on current and time and open-circuit voltage, covering a wide range of machining powers and per-pulse energies. The machining performances were evaluated in terms of the MRR, TMRR, and TWR, while the SQ was estimated according to the roughness values (Ra, Rz) and the average white layer thickness. Moreover, the characteristics of the WL were further studied through optical and SEM microscopy, whilst EDX and micro-hardness measurements were also carried out, allowing a more comprehensive and detailed analysis of the WL. For all the aforementioned performance indexes, i.e., MRR, TMRR, TWR, Ra, Rz, and AWLT, ANOVA was performed, and finally, by adopting the GRA method a multi-objective optimization was proposed.

The most notable conclusions deduced from the current study are:

- The MRR is mainly affected by the pulse-on current, while the pulse-on time and the open-circuit voltage have a minor and vague impact on MRR. Additionally, for the low pulse-on currents (5 and 7 A) the MRR remains almost stable for all the machining parameter combinations.
- The TMRR is mainly affected by the combination of the machining parameters and not as a direct result of a specific change in the machining parameters.
- The lowest TWR was measured for the higher pulse-on times (i.e., 50 and 100 μs), while it was also almost constant regardless of the other machining conditions (i.e., pulse-on current and open-circuit voltage).
- The roughness values (Ra, Rz) mainly increase as the pulse-on time and current increase, although an increase of the open-circuit voltage reduces the surface roughness.
- The AWLT values significantly deviate for the lower open-circuit voltages depending on the machining parameters combination. The lowest WL thickness and with minimum deviation was measured for the higher Vos (i.e., 160 and 200 V), a result that can be ascribed to the capability of more efficient flushing and thus better molten material removal.
- The WL has over 400% increased micro-hardness compared with the bulk material. This increase in the WL micro-hardness is mainly attributed to the material’s amorphization since, according to EDX maps, no change in the material’s chemical composition occurred.
- The machined surfaces are covered by craters, whose central area is smooth, and their rim is made up of bulky formations. Moreover, pockmarks, microcracks, microporosity and voids are observed to a different degree depending on the machining conditions.
- By employing the GRA, a multi-objectives optimization can be achieved, even for performance indexes that are competitive (i.e., MRR, TWR, and Ra). Nevertheless, it is substantial that only the by case absolutely necessary performance indexes should be considered in order for a clear result tο emerge.
- More specifically, according to the GRG grades during the optimization, in order to achieve better TWR-MRR-Ra, the optimal combination of parameters is 17 A, 50 μs, and 120 V, although when we considered the AWLT, the optimal parameters decreased the pulse-on time and current (5 A, 50 μs, and 160 V).

## Figures and Tables

**Table 1:** CALMAX chemical analysis and thermophysical properties.

**Typical Analysis %**	**Fe**	**C**	**Si**	**Mn**	**Cr**	**Mo**	**V**
	bal.	0.6	0.35	0.8	4.5	0.5	0.2
**Physical Properties**							
Density [kg/m^3^]		7770					
Thermal Conductivity [W/mK]		27					
Specific Heat [J/kgK] at 293 K–473 K–679 K		455–525–608					

**Table 2:** Experimental parameters.

Machining Conditions	Level 1	Level 2	Level 3	Level 4
Discharge current I_p_ [A]	5	9	13	17
Pulse on-Time T_on_ [μs]	12.8	25	50	100
Open-circuit voltage V_o_ [V]	80	120	160	200
Close circuit voltage V_c_ [V]	30			
Duty Factor	0.5			
Dielectric	Synthetic hydrocarbon fluid			
Dielectric Flushing	Side flushing with pressure			
Dielectric Flushing Pressure [MPa]	0.7 (Constant under the whole conditions)			

**Table 3:** Control parameter combinations based on the Taguchi L_16_ DOE.

# EXP	I_p_ [A]	T_on_ [μs]	V_o_ [V]	# EXP	I_p_ [A]	T_on_ [μs]	V_o_ [V]
1	5	12.8	80	9	13	12.8	160
2	5	25	120	10	13	25	200
3	5	50	160	11	13	50	80
4	5	100	200	12	13	100	120
5	9	12.8	120	13	17	12.8	200
6	9	25	80	14	17	25	160
7	9	50	200	15	17	50	120
8	9	100	160	16	17	100	80

**Table 4:** Experimental results.

# EXP	MRR [mm^3^/min]	TMRR [mm^3^/min]	TWR	Ra [μm]	Rz [μm]	RSk	Rku	AWLT [μm]
1	0.337	0.119	0.354	1.37	7.02	−0.52	3.34	3.68
2	0.946	0.423	0.448	2.56	15.23	−0.29	3.84	14.98
3	0.427	0.014	0.033	2.25	16.15	0.3	2.73	4.30
4	0.303	0.007	0.022	2.56	15.77	−0.27	2.97	7.72
5	1.291	0.244	0.189	2.33	17.73	−0.18	2.64	4.26
6	0.754	0.201	0.267	2.39	12.49	0.57	2.6	4.41
7	1.035	0.074	0.071	2.28	12.31	0.59	3.71	7.15
8	0.722	0.047	0.065	2.93	17.24	0.17	3.13	7.61
9	5.424	1.855	0.342	3.59	18.92	−0.1	2.58	3.31
10	3.925	0.391	0.100	3.77	18.90	0.29	3.23	5.21
11	5.517	0.374	0.068	4.75	26.73	0.34	2.99	14.25
12	4.351	0.133	0.031	4.67	23.97	1.07	4.77	9.87
13	7.032	2.330	0.331	2.69	17.58	0.59	2.95	7.38
14	5.513	1.184	0.215	3.22	22.27	0.18	2.73	7.64
15	7.979	0.799	0.100	6.13	34.67	0.3	2.95	6.35
16	4.031	0.414	0.103	5.63	31.74	0.46	3.25	13.50

**Table 5:** Results of the grey relational analysis.

Machining Parameters			Grey Relational Coefficients				Grey Relational Grades		
I_p_[A]	T_on_ [μs]	V_o_ [V]	MRR	TWR	Ra	AWLT	MRR TWR	MRR TWR–Ra	MRR–TWR Ra–AWLT
5	12.8	80	0.334	0.391	1.000	0.940	0.363	0.575	0.666
5	25	120	0.353	0.333	0.666	0.333	0.343	0.451	0.422
5	50	160	0.337	0.952	0.730	0.855	0.645	0.673	0.719
5	100	200	0.333	1.000	0.667	0.570	0.667	0.667	0.642
9	12.8	120	0.365	0.561	0.712	0.860	0.463	0.546	0.624
9	25	80	0.347	0.465	0.700	0.841	0.406	0.504	0.588
9	50	200	0.356	0.813	0.723	0.603	0.584	0.631	0.624
9	100	160	0.346	0.834	0.604	0.576	0.590	0.595	0.590
13	12.8	160	0.600	0.400	0.517	1.000	0.500	0.506	0.629
13	25	200	0.486	0.734	0.498	0.754	0.610	0.572	0.618
13	50	80	0.609	0.824	0.413	0.348	0.716	0.615	0.549
13	100	120	0.514	0.962	0.419	0.471	0.738	0.632	0.591
17	12.8	200	0.802	0.408	0.643	0.589	0.605	0.618	0.610
17	25	160	0.609	0.525	0.563	0.574	0.567	0.565	0.568
17	50	120	1.000	0.732	0.333	0.657	**0.866**	**0.688**	0.681
17	100	80	0.493	0.726	0.358	0.364	0.609	0.526	0.485

## Data Availability

Data available on request from the corresponding author.

## Nomenclature

EDM	Electrical Discharge Machining	-
AWLT	Average White Layer Thickness	μm
*E_fin_*	Electrode weight after machining	gr
*E_st_*	Electrode weight before machining	gr
HAZ	Heat Affected Zone	μm
I_p_	Pulse-on current	A
MRR	Material Removal Rate	mm^3^/min
Ra	Mean Roughness	μm
Rz	Maximum peak to valley height	μm
SCD	Surface Crack Density	m/mm^2^
SQ	Surface Quality	-
ST	Surface Topography	-
TMRR	Tool Material Removal Rate	mm^3^/min
T_on_	Pulse-on time	μs
TWR	Tool Wear Ratio	%
*t_mach_*	Mahining time	min
*W_fin_*	Workpiece weight after machining	gr
*W_st_*	Workpiece weight before machining	gr
WL	White Layer	-
*ρ_el_*	Electrode density	gr/mm^3^
ρ_w_	Workpiece density	gr/mm^3^
*Γ*	Grey Relational Grades	-
*γ*	Grey Relational Coefficients	-
